# Treatment of children and adolescents with avoidant/restrictive food intake disorder: a case series examining the feasibility of family therapy and adjunctive treatments

**DOI:** 10.1186/s40337-018-0205-3

**Published:** 2018-08-03

**Authors:** Wendy Spettigue, Mark L. Norris, Alexandre Santos, Nicole Obeid

**Affiliations:** 1 Department of Psychiatry, Children’s Hospital of Eastern Ontario, University of Ottawa, 401 Smyth Road, Ottawa, ON K1H 8L1 Canada; 2 Division of Adolescent Health, Department of Pediatrics, Children’s Hospital of Eastern Ontario, 401 Smyth Road, Ottawa, ON K1H 8L1 Canada; 3 Children’s Hospital of Eastern Ontario Research Institute, 401 Smyth Road, Ottawa, ON K1H 5B2 Canada

**Keywords:** Avoidant/restrictive food intake disorder, Family therapy, Pharmacotherapy, Case series

## Abstract

**Background:**

To date, little research has examined the effectiveness of either modified Family-Based Therapy or psychopharmacological treatments for patients diagnosed with avoidant/restrictive food intake disorder (ARFID), and there is little evidence to guide clinicians treating children and adolescents with ARFID. This case series describes the clinical presentations, treatments and outcomes of six patient diagnosed with ARFID who were treated sequentially by a child psychiatrist and adolescent medicine physician in a hospital-based eating disorder program.

**Case Presentations:**

Five out of six cases were female and median age of patients at assessment was 12.9 (*SD* = 1.13) years. On average, patients’ percentage of treatment goal weight was 80.5% at initial assessment (*SD* = 8.56) and 81.9% (*SD* = 7.08) when family therapy began. Cases 1, 2 and 3 were admitted to a specialized inpatient unit at assessment due to medical instability (2) or failed outpatient treatment (1), and all six cases presented with severe co-morbid anxiety. All patients were treated using a combination of medical monitoring, family therapy, medication (including olanzapine, fluoxetine and in two cases cyproheptadine), and cognitive behavioural therapy. At treatment termination, all six patients had achieved their goal weight.

**Conclusion:**

These cases illustrate the complex ways in which young patients with ARFID can present, the illness’ effect on development and mental health, and the positive outcomes associated with weight gain and concurrent treatment for co-morbid anxiety disorders.

## Plain English summary

To date, little research has examined the effectiveness of either family therapy or medications for patients diagnosed with avoidant/restrictive food intake disorder (ARFID), and there is little evidence to guide clinicians treating children and adolescents with this newly classified eating disorder. This case series describes six patients diagnosed with ARFID and treated by a child psychiatrist and a pediatrician in a hospital-based eating disorder program. Five out of six cases were female and average age of patients was 12.9 years, with an average percentage of goal weight at assessment of 80.5%. Three cases were admitted to a specialized inpatient unit at assessment due to medical instability or failed outpatient treatment, and all six cases presented with severe anxiety. All patients were treated using a combination of medical monitoring, family therapy, medication and cognitive behavioural therapy. At treatment termination, all six patients had achieved their goal weight. These cases illustrate the complex ways in which young patients with ARFID can present, the illness’ effect on development and mental health, and the positive outcomes associated with weight gain and concurrent treatment for anxiety.

## Background

Family-based therapy (FBT) is a treatment method that has been robustly used for the treatment of eating disorders (EDs) in adolescents, such as anorexia nervosa (AN) and bulimia nervosa (BN) [1–4]. Early models of FBT were first introduced in the 1970’s and 80’s [5, 6], providing a foundation for the modern-day Maudsley outpatient FBT model, which was manualized and popularized by Lock et al. in *Treatment manual for anorexia nervosa: a family-based approach* [7]. FBT is designed to empower family members to support the recovery of the patient in a home setting by providing parents and siblings with education on EDs, lifting blame and guilt from the family and patient, raising anxiety about the seriousness of the illness, externalizing the ED as an enemy, and encouraging parents to implement refeeding strategies [8]. Meta-analytic research has shown that FBT is more effective at increasing rates of remission when compared to treatment as usual in adolescent patients suffering from AN or BN [2, 3]. Further research has suggested that FBT is effective at promoting weight gain and improving psychological functioning in AN patients [1].

Another line of evolving research suggests that olanzapine, an atypical antipsychotic, may also have merit as an adjunctive medication for patients with AN, with reported improvements in weight gain, anxiety and body image, although few adolescent-specific studies have been conducted to date [9–13]. To a similar degree, some conflicting research has shown that the use of fluoxetine, a selective serotonin reuptake inhibitor (SSRI), may reduce co-morbid symptoms of depression, anxiety, and obsessive compulsive disorder for weight-restored adult AN patients [14–16]. SSRIs are indicated for the treatment of anxiety and depression in adolescents without eating disorders [17, 18]. Despite the evidence supporting the use of FBT as an effective treatment for AN and BN in children and adolescents, and evidence for the possible role of both olanzapine and fluoxetine in the treatment of youth with AN, to date, little research has examined the effectiveness of family therapy or psychopharmacological treatments for patients diagnosed with avoidant/restrictive food intake disorder (ARFID).

Introduced in the 5th edition of the Diagnostic and Statistical Manual of Mental Disorders (DSM-5) [19], ARFID is a type of restrictive ED characterized by a persistent failure to meet appropriate nutritional and/or energy needs, resulting in at least one of the following: significant weight loss, significant nutritional deficiency, dependence on enteral feeding or nutritional supplements, and/or a marked interference in psychosocial functioning. Further, the disturbance is not explained by a lack of food availability, cultural practices, body image concerns, or concurrent medical and/or mental conditions. Due to its recent introduction and heterogeneous nature, specialized treatments for patients diagnosed with ARFID have not yet been well established [20, 21].

A recently published review of 48 ARFID cases by Norris and colleagues [20] suggests substantial heterogeneity in terms of case presentations and contributing factors. Three specific subtypes of ARFID were identified (aligning with example presentations outlined in the DSM-5): those with weight loss and/or medical compromise as a consequence of apparent low appetite and lack of interest in eating (classified as “ARFID-limited intake” subtype), restriction arising as a result of sensory sensitivity i.e. “picky eaters” (classified as “ARFID-limited variety” subtype), and restriction based upon food avoidance and/or fear of aversive consequences of eating, such as fear of pain, nausea or choking (“ARFID-aversive” subtype). Clinical characteristics of the 48 patients varied depending on the assigned subtype; we hypothesize that recognizing and understanding these differences will have relevance as to the utility and success of specific targeted treatments for ARFID. There was some overlap of ARFID subtypes, with 13% of the sample  presenting with a mixed presentation. The aim of this case series is to clinically describe and examine immediate outcomes in the treatment of six adolescent ARFID patients sequentially treated by a child psychiatrist and adolescent medicine (AM) physician in a tertiary care hospital ED setting and to comment on how each case presentation helped to influence the overall treatment plan. Of our six cases, two represent the “ARFID-aversive” subtype and the other four represent mixed subtypes: one with a combination of “ARFID-limited variety” subtype and “ARFID-aversive” subtype, and three with mixed “limited variety” and “limited intake” subtypes. All six patients received family therapy sessions that were based on the ‘Maudsley’ model of FBT principles for treating adolescent AN, but modified and adapted to the particular patient, diagnosis, and subtype of ARFID; these ‘modified FBT’ sessions will subsequently be referred to in the paper as “family therapy” sessions (though note they are referred to on the patient weight graphs below as “FBT” sessions due to limited space).

## Method

A retrospective review was conducted of the medical charts of six sequential patients with ARFID referred for treatment by an Adolescent Medicine (AM) physician based at a Canadian tertiary care center to a child psychiatrist on an adolescent ED team at the same center, and information gathered was subsequently de-identified and stored in an electronic database. For confidentiality purposes, names included in this paper have been changed to protect patients’ privacy. Signed consent and assent forms were obtained from patients and their primary caregivers for the study, which was approved by the hospital’s Research Ethics Board.

## Case presentations

Table 1 provides an overview of demographic information for the 6 patients included in this case series. On average, patients’ percentage of treatment goal weight (%TGW), was 80.5% at initial assessment (*SD* = 8.56) and 81.9% (*SD* = 7.08) when family therapy began. TGW for each patient was calculated by the AM physician using established methods for ED populations (in these cases by using prior premorbid growth percentiles to establish the TGW) [22]. The median age of patients at treatment initiation was 12.9 (*SD* = 1.13) years. Three of the six patients were admitted to a specialized ED inpatient unit at initial assessment due to medical instability (2) or due to failed outpatient treatment prior to family therapy (1). At the termination of treatment with the ED team, all six patients achieved their TGW.

**Table 1:** Demographic Information of Described ARFID Patients

	ARFID Cohort (*N* = 6)	Range (min – max)
Sex		
Male	1 (17%)	
Female	5 (83%)	
Ethnic Background		
Caucasian	6 (100%)	
Age [Mean (*SD*)]	12.73 (1.15)	10–14
Length of Self-reported Illness at Initial Assessment [Mean (*SD*)]	14.67 (10.42)	3–24

*Notes.* Where appropriate, data are expressed as mean (SD) or n (%). Age is expressed in years; length of illness is expressed in months

### Case 1: Amy

Amy is a 12.5 year old girl in grade 7 with a history of mild phobias but no previous social anxiety, who lives with two working parents in a middle class home. At 11 years of age she became terrified after witnessing her dog choke on a piece of rawhide bone. Thereafter, she started to worry obsessively about choking. She started to prefer soft foods, to take tiny bites and to chew her food many times before she would swallow. She took a very long time to eat, wouldn’t eat lunch or snacks at school, and became increasingly slow and restrictive in her eating. Amy was admitted to hospital at a body mass index (BMI) of 13.5 kg/m^2^ (83.0% of TGW) and diagnosed with ARFID. Her case history was most in keeping with the ARFID-aversive subtype. She spent 38 days in hospital, receiving family therapy and psychoeducation and support for parents, who were very resistant to, and anxious during, passes out of the hospital environment. With inpatient family therapy sessions, parents were empowered to take control of nutrition on passes and to ensure that Amy finished everything. Her treatment was initially augmented in hospital with olanzapine 2.5 mg at bedtime, and as she approached her TGW she was started on fluoxetine 10 mg/day to help manage her severe anxiety.

Amy was discharged home at 100% of her TGW, but with an ongoing fear of choking that continued to impact her eating (e.g. still taking very small bites and a long time to eat, and avoiding foods that she feared she might choke on). Outpatient family therapy was augmented with cognitive behavioural therapy (with parents present). Amy developed and worked on a hierarchical ladder of feared foods, and was encouraged to practice eating using a timer, to take bigger bites, and to eat with peers at school. Although Amy did not describe social anxiety at school or worrying about schoolwork, she did develop a fear of going out with parents. Her fluoxetine was increased to 20 mg/day as a consequence of Amy’s increased anxiety. The family described the increased dose of fluoxetine as helping significantly with Amy’s fear of going out, and opted not to increase the dose further. Over the next 5 months (and nine therapy sessions), Amy recovered completely from her ED and fear of choking and was able to reach a healthy weight and to continue to grow, at which point she was discharged from care from the ED team.

Weight at start of family therapy: 30.3 kg.

%TGW at start of family therapy: 83%.

Weight at end of family therapy: 43.2 kg.

**Fig. 1:**
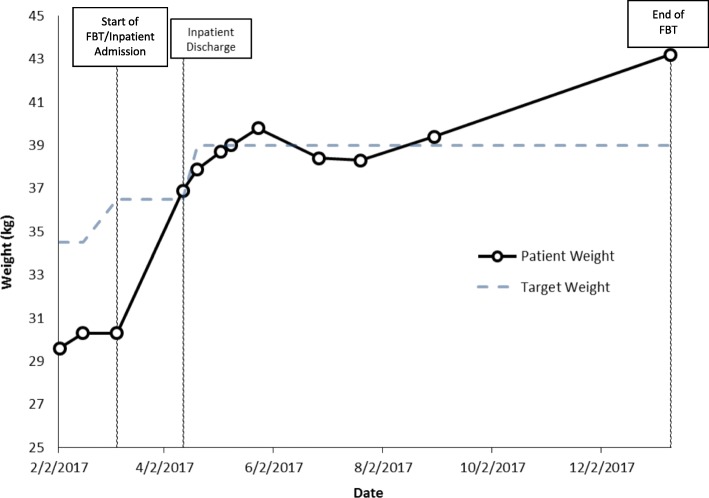
Case 1 weight graph (Amy)

%TGW at end of family therapy: > 100% (Fig. 1).

### Case 2: Susan

Susan is a 10.9 year old girl who lives at home with 2 professional parents and a younger sister. She was described as a very anxious child; past medical history was notable for a history of frequent stomach pains of no known medial cause, and school refusal, though no eating or growth problems. Susan developed repeat episodes of viral gastroenteritis over a two week period which left her convinced that resuming eating had caused her gastro-intestinal symptoms. As a result, over the next few months she progressively ate less and lost weight. She underwent a full medical work-up but no pathology was identified. Her parents progressively eliminated foods that could potentially exacerbate her symptoms of abdominal pain and nausea, but with limited effect. She was admitted to the pediatric ward weighing 75.8% of TGW (BMI 11.8 kg/m^2^). Given her refusal to eat, she was initially re-nourished with liquid nutrition (Ensure) via nasogastric (NG) tube, but weight gain was very slow and difficult. She refused to eat or drink, would kick and scream and become hysterical whenever food was presented, and screamed throughout the duration of her NG feeds. One month after admission there had been minimal progress, so the ED team was consulted and family therapy was initiated. Her case history was felt to be in keeping with ARFID-aversive subtype.

In addition to family therapy, Susan was treated with olanzapine over the course of her admission to help with her severe agitation and anxiety; she was started on 2.5 mg at night and the dose was gradually increased to a maximum of 2.5 mg in the morning and 5 mg at night.

Both parents were convinced that this must be a medical problem. The therapist worked to empower and educate parents, lift guilt and blame, and also to raise anxiety about the need for parents to take control of the nutrition and help their daughter to eat. The therapist explained the ‘vicious cycle’ of how weight loss leads to more anxiety, a ‘smaller’ stomach, more stomach pain, and the need for more nutrition to reach IBW. Thus, the only ‘way out’ is with renourishment and weight gain. Parents would try to be firm and help Susan to take the nutrition, but Susan would kick and scream hysterically and refuse, crying that her stomach hurt too much and she couldn’t eat. Parents and Susan were helped to see that “not eating is not an option;” it is a mandatory part of treatment and the only way to recover. Susan was taught to use relaxation techniques and her mother was taught to support her, e.g. by providing soothing massage, empathy, and whatever helped Susan to tolerate taking the nutrition. Susan insisted that all that helped was walking. Thus, she would take her (mandatory) nutrition, crying and sobbing loudly, and then walk up and down the hospital halls. Gradually Susan’s intake increased, along with her weight, and the pain and hysterical sobbing lessened. Parents were encouraged to take Susan on as many passes as possible, which she initially resisted, saying that it hurt too much to eat at home. She and her parents were helped to see that change is stressful and that unless Susan was going to live in the hospital forever, she would need help with learning to eat at home. Gradually, passes became increasingly more successful, and weight increased. Susan was discharged home two months after admission, at 100% of her initial TGW.

Following discharge, Susan continued weekly family therapy sessions. Although her weight gain continued as she grew in height, she resisted numerous foods that she was fearful of eating for fear of abdominal pain. She also exhibited separation anxiety and school avoidance. Family therapy continued, cognitive behavioural therapy was also utilized, and gradually, food variety increased. Parents opted for home schooling. Fluoxetine 10 mg in the morning was started for severe anxiety, and the dose was titrated over the following months up to 40 mg/day. Concurrently, her olanzapine was tapered and discontinued. At the end of treatment (after 6 therapy sessions but with follow up by the AM physician over a total of 3.5 months), she was able to eat all foods, she could socialize and participate in sports, and she could eat without issue at restaurants (which previously caused anxiety as well).

Weight at start of family therapy: 26.7 kg.

%TGW at start of family therapy: 79%.

Weight at end of family therapy: 41.6 kg.

**Fig. 2:**
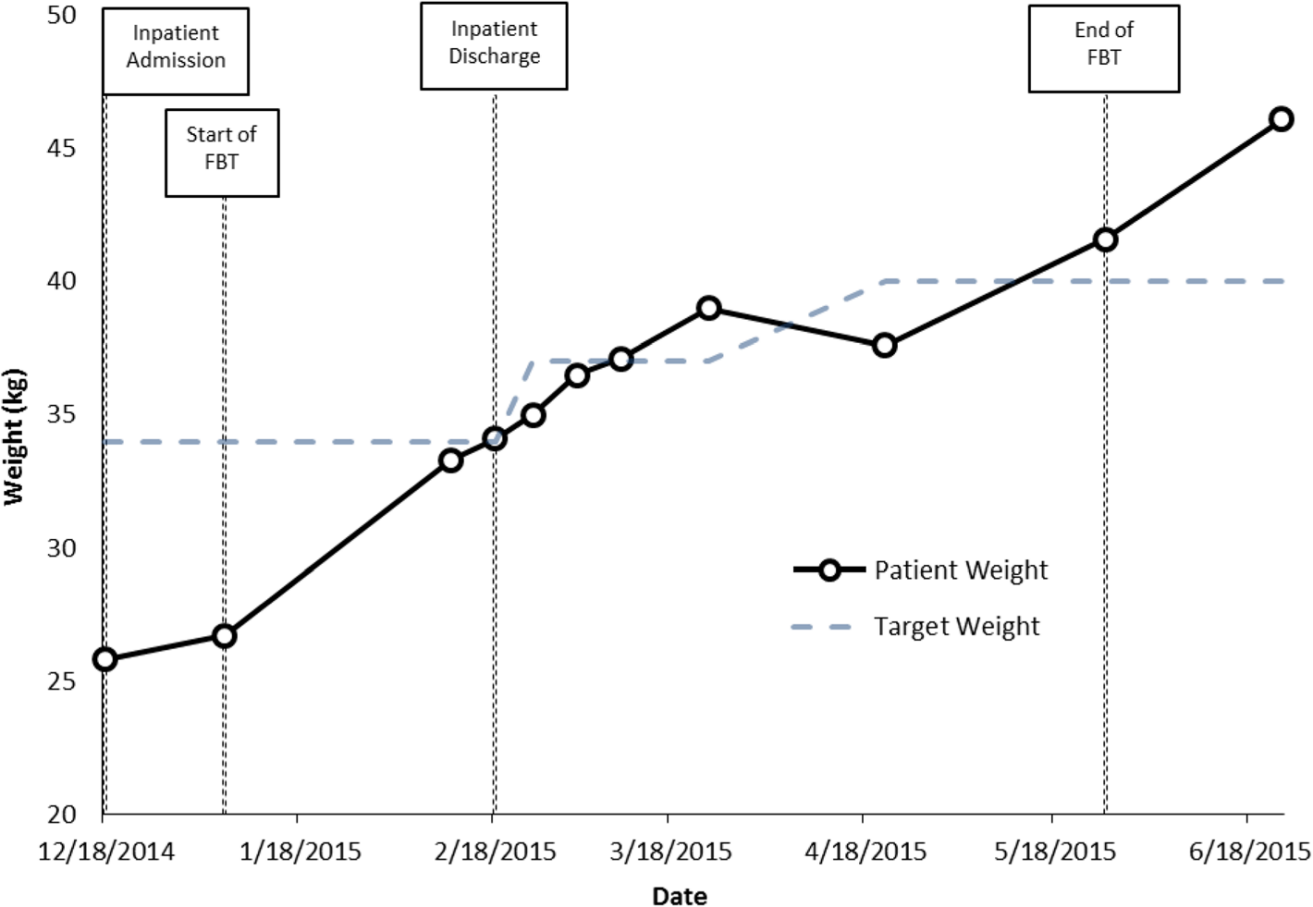
Case 2 weight graph (Susan)

%TGW at end of family therapy: > 100% (Fig. 2).

### Case 3: Ethan

Ethan is a 13.1 year old boy with mild-moderate autism spectrum disorder (ASD) who lives on a farm with his father and grandparents. He enjoys school, where he attends a special class because of his intellectual disability. He had a history of “picky” eating but no history of low weight or growth problems. One day while riding, Ethan fell off his horse and injured his ribs. He experienced severe rib pain with swallowing. As a result, he limited his food intake to minimize the pain. As weight decreased, his restriction intensified and he became increasingly anxious about eating. Ethan was eventually admitted to hospital where he spent one month on the pediatric ward, had a full medical work-up, and was discharged home at a slightly lower weight than at admission. His history was in keeping with a mixed ARFID presentation: ARFID-limited variety subtype plus ARFID-aversive subtype. He was readmitted to a specialized ED inpatient unit in the weeks that followed (at 72% TGW). Father needed to work on the farm, so Ethan’s grandmother stayed in hospital with Ethan and worked with the family therapist.

Initially, Ethan would take very small amounts orally and then say he was too “full”. He would have temper tantrums when pushed to eat more, and would often gag or vomit if made to eat. His treatment was augmented with olanzapine and titrated from 2.5 mg up to 7.5 mg/day to help with his anxiety around meals, and to facilitate weight gain. Given his longstanding food selectivity and sensory issues, the ED dietician also allowed accommodations with respect to the meals that he received in hospital. Grandmother was empowered to ensure that Ethan finished everything on his tray, and was asked by staff to tell Ethan that if he didn’t finish his meal he could not have a pass off the ward. However, grandmother said she did not want to be the “bad guy” and instead chose (and was empowered to choose) to find other ways to help Ethan to finish his meals. For example, she chose to take him in a wheelchair for a walk around the hospital after he had finished half his meal, and they would then return and he would finish the rest of his meal. This worked well. Father would visit on weekends to provide meal support, and despite his anxiety about doctors and hospitals, started attending family sessions. Ethan was thrilled to be granted passes to eat at fast-food restaurants, but his weight started to drop as passes increased. He was ordering high calorie foods, but would get up and leave the restaurant before he had finished his portion. The therapist worked with both father and grandmother to help them to support Ethan to stay and finish all of his nutrition before they would leave.

Ethan’s pace, intake and weight gradually improved and he was discharged after a two month stay at 88% of his TGW so that he could begin the new school year. He was discharged on olanzapine 5 mg at bedtime and fluoxetine 10 mg/day. The family continued outpatient family therapy over the next month, but weight dropped and the family was resistant to driving the long distance to the hospital for therapy sessions. The therapist followed up with grandmother by phone and empowered her to track Ethan’s weight at home and to increase nutrition in keeping with his increased activity on the farm. His fluoxetine was gradually titrated up to 40 mg/day over the next three months. The fluoxetine was noted to significantly decrease his anxiety at school, and weight gain improved thereafter. At a follow-up appointment 4 months after discharge, Ethan had reached his TGW. His olanzapine was tapered and discontinued very slowly over the following months and he continued on fluoxetine with excellent effect.

Weight at start of family therapy: 31.8 kg;

%TGW at start of family therapy: 72%;

Weight at end of family therapy: 37.6 kg;

%TGW at end of family therapy: 85% (though some telephone conversations continued).

**Fig. 3:**
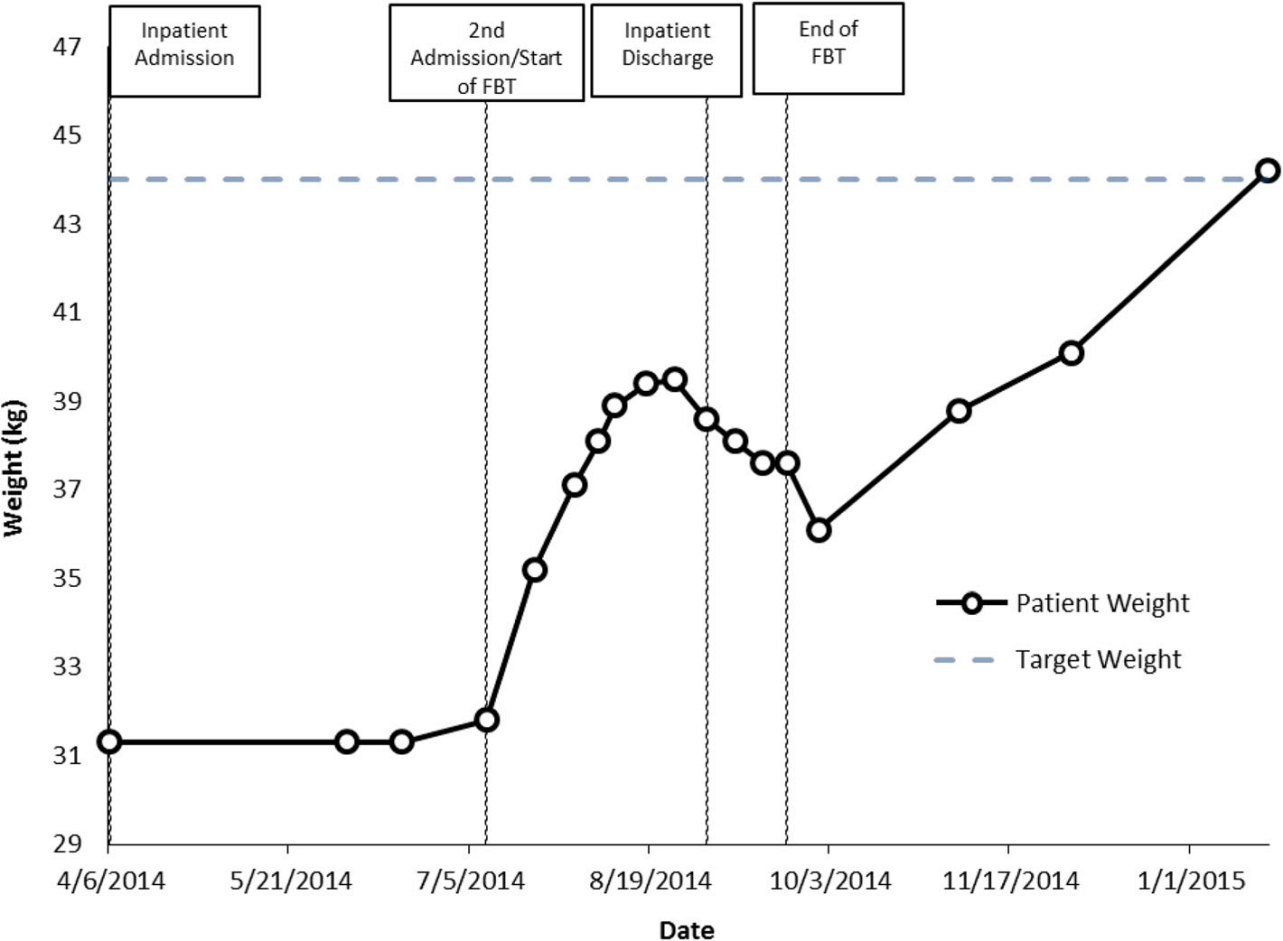
Case 3 weight graph (Ethan)

Weight at last follow up with AM physician: 44.2 kg (100% TGW) (Fig. 3).

### Case 4: Jacqueline

Jacqueline is a 14.4 year old girl with a history of social and generalized anxiety disorder, obsessive compulsive disorder (OCD) and ASD traits. She presented with a 1–2 year history of increasingly restrictive intake. Parents noted: “She has always been picky, but if you gave her the foods she liked she would eat them; now she won’t eat them.” Parents described getting every bite into her as “a struggle.” They also described her as very rigid, controlling, and more recently having huge temper tantrums, sleep problems and problems concentrating. She also looked and acted like a much younger child, and had symptoms of what appeared to be severe attention-deficit hyperactivity disorder (ADHD), as she frantically ran around the office and refused to sit still during the consultation, although she was described as a good student until this year, with no prior history of ADHD or of school or behavior problems. At initial assessment, she was on the 3rd percentile for weight; this put her at 90% of her TGW based on her growth curve, but with a history of having been underweight for a prolonged period and having always been a picky eater with very low appetite. She was in grade 9 at the time of assessment with a history of social isolation and increased anxiety at school for the past two years. There was a past history of bullying and social isolation in elementary school but no recent acute stressors or trauma. Her history was most consistent with a mixed ARFID presentation: ARFID-limited intake and ARFID-limited variety subtypes. A comprehensive medical work-up was negative.

Family therapy (with both divorced parents attending with Jacqueline) began within weeks of her initial assessment, and her treatment was augmented with olanzapine 2.5 mg at bedtime, which was later increased to 5 mg. The parents opted not to use a weight graph in sessions, as they were worried that Jacqueline would become “obsessed” with the numbers, but parents were informed weekly whether weight was up or down. Jacqueline was kept home from school and her mother stayed home from work to focus on renourishing her. After a few weeks Jacqueline’s weight had improved and she became bored and expressed a desire to return to school, so attended school in the mornings in the “resource room” (where she ate a ‘mandatory’ morning snack) and then came home for lunch. Jacquie gained weight well as her treatment continued over the course of 6 months (13 family therapy sessions). Of note, as weight increased, she became less “picky” about her eating and able to eat more variety. She also became significantly less hyperactive and regressed, and began to speak more in therapy. As a result, her treatment was augmented with weekly individual therapy sessions in addition to her family therapy. She expressed very high anxiety, both about her schoolwork and about socializing with peers. She was given information about ‘Asperger syndrome,’ which she found helpful. She continued to gain weight and reached her TGW, ending up on the 25th percentile for weight, with a height near the 25th percentile and an overall BMI of 50% for girls her age. Once she reached her TGW, the olanzapine was tapered and stopped, and an SSRI (fluvoxamine 25 mg/day) was started to target her anxiety. The fluvoxamine was increased gradually from 50 mg to 100 mg/day, but this dose was associated with increased agitation and restlessness, including one weekend when Jacqueline spent the entire weekend in her pajamas pacing back and forth in her bedroom. The fluvoxamine was decreased back down to 50 mg/day and the agitation disappeared, although the family chose to continue the medication as they found the 50 mg dose continued to have a positive effect on her anxiety. By the end of treatment, Jacqueline could sit well throughout a one-hour therapy session with no symptoms of her initial hyperactivity or restlessness; she was also less irritable, more cheerful and affectionate with her family, and more mature and articulate.

Weight at start of family therapy: 37.4 kg.

%TGW at start of family therapy: 93%.

**Fig. 4:**
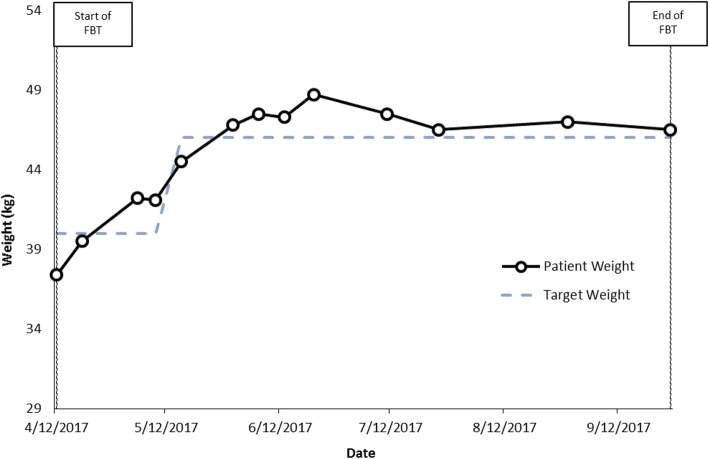
Case 4 weight graph (Jacqueline)

Weight at end of family therapy: 46.5 kg;

%TGW at end of family therapy: > 100% (new TGW: 46 kg) (Fig. 4).

### Cases 5 and 6: Hannah and Emily

Hannah and Emily are non-identical twins, 12.9 years old in grade 7 at the time of assessment. Both presented at low weight: Hannah was 73.2% of TGW and Emily was 83.3% of TGW. Emily had a history of severe anxiety and OCD; Hannah was described as not anxious by parents, although her anxiety became obvious over time, once she was re-nourished. The twins were very close to each other. Both girls appeared young for age, and had separation anxiety, and both were strongly attached to their mother, who they slept with every night. Family history was notable for anxiety, bipolar disorder and schizophrenia. Both girls were described as having always been highly picky eaters with long-standing low appetites; mother described being very stressed by always having to cook a different supper for each girl. Both patients were felt to exhibit symptoms most consistent with a mixed ARFID presentation: ARFID-limited intake and ARFID-limited variety subtypes.

During family therapy sessions, which focused on food intake, weight was graphed weekly and parents were empowered to do whatever they needed to do to increase weight for both girls. Parents opted to add liquid supplements twice a day to help improve overall intake. Both girls tried hard to please, but described low appetite, difficulty eating enough food, and not liking many foods. For example, Emily described eating just macaroni salad, goldfish crackers, water and Oreo cookies all day. Weight gain was slow, so treatment was augmented with cyproheptadine (an appetite stimulant, which was started at a dose of 2 mg twice a day and increased to 2 mg in the morning and 4 mg in the afternoon) and then subsequently olanzapine was added as well, at a dose of 2.5 mg at bedtime (the dose was not increased beyond this as their mother was worried that the girls might have trouble waking in the morning). For both girls, olanzapine helped with sleep, anxiety and weight gain. It was clear that Emily’s anxiety was very high at school, which interfered with her appetite and intake and was also associated with low mood and self-harm urges, so parents elected to take her out of school. This was helpful, and as Emily’s weight was increasing at a faster rate, Hannah was then also taken out of school.

**Fig. 5:**
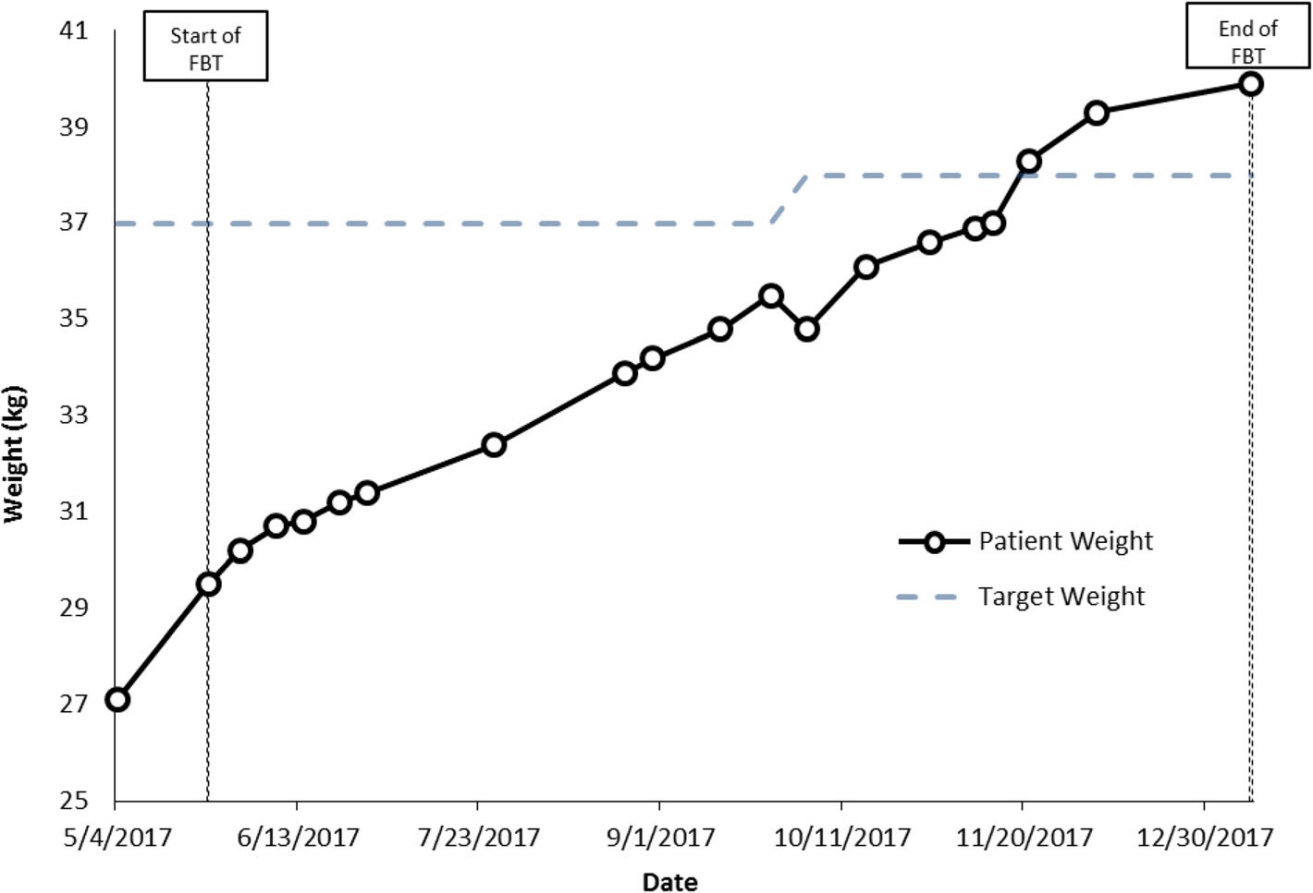
Case 5 weight graph (Hannah)

**Fig. 6:**
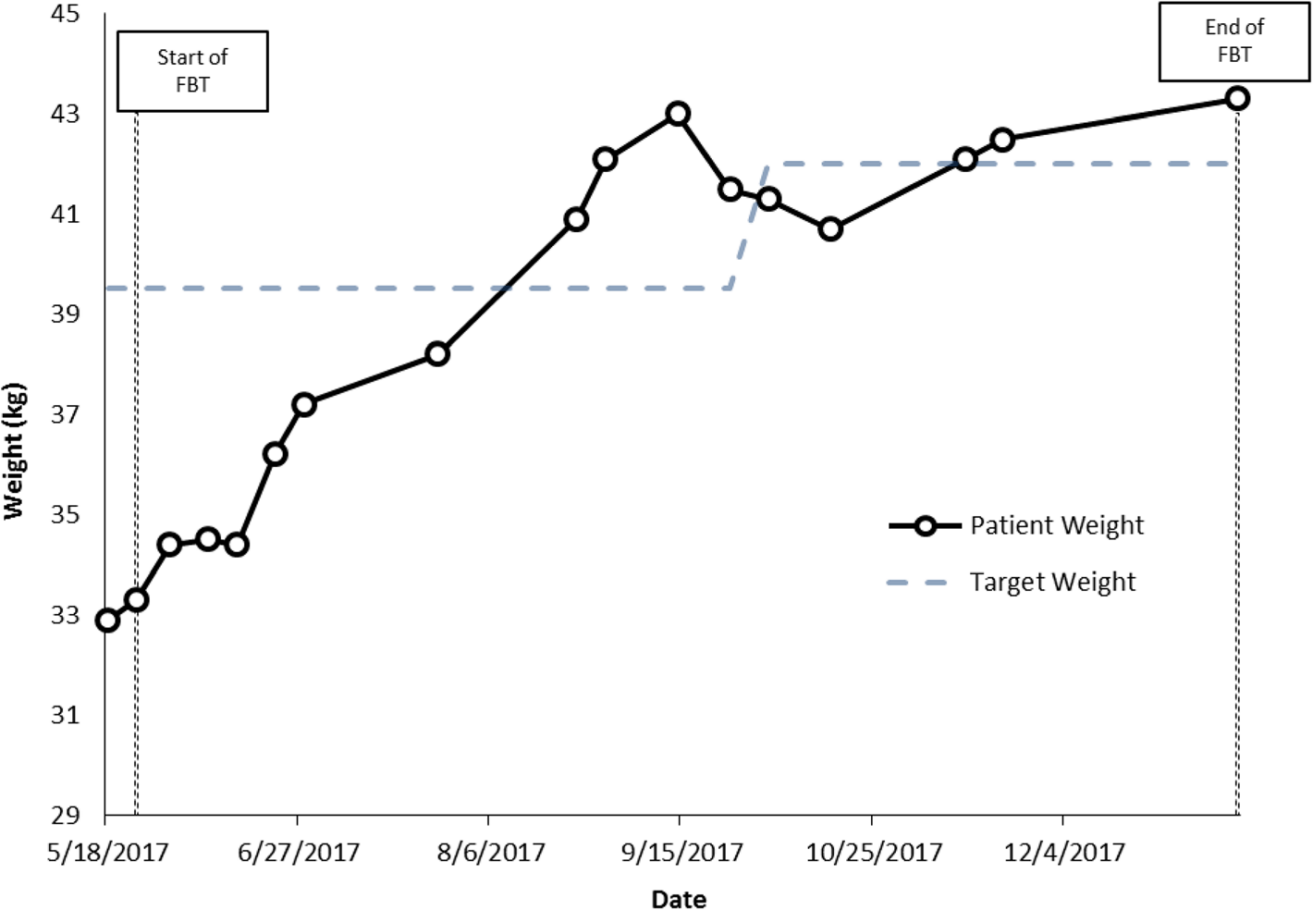
Case 6 weight graph (Emily)

As weight increased, social and generalized anxiety did not decrease for either girl, but food variety did improve. Both twins started eating and liking new foods, and by mid-Fall were back at school and able to eat the same thing for supper for the first time in their lives. In each case, as the girls approached their TGW and started to grow taller as well, fluoxetine was prescribed to help target their anxiety (starting at 10 mg/day and gradually increased to a dose of 40 mg/day) and the cyproheptadine was tapered and eventually stopped. The olanzapine 2.5 mg at bedtime was also stopped, but both girls experienced a significant increase in social and generalized anxiety after the olanzapine was discontinued, so it was restarted, and eventually the dose was increased to 3.75 mg at bedtime due to ongoing severe anxiety. Emily reached her TGW at family session 8 and Hannah at session 16 (within 3 and 6 months of treatment respectively). During treatment, Hannah’s weight went from 27.1 kg at assessment to 39.9 kg at last follow up, while Emily’s weight increased from 32.9 kg at assessment to 43.3 kg at follow up (Figs. 5 and 6).

## Discussion

ARFID is a new diagnosis in the DSM-5 [19]. While the literature describing ARFID is increasing [20, 21, 23–27], to date, there are no randomized or open trials examining treatment outcomes for ARFID, and no treatment guidelines for clinicians to follow. Adapted FBT has been suggested as a potentially useful treatment for pediatric ARFID patients [28, 29]. Although recommended to a similar degree when compared to patients with AN in the study by Strandjorn and colleagues [29], few adolescent ARFID patients took part in the FBT sessions, thus limiting the study’s conclusions on the utility of adapted FBT as an integrative treatment method for ARFID. Furthermore, no accounts of barriers and/or facilitators to implementing adapted FBT in this population have been reported.

Similarly, currently there is only one case series that explores the utility of olanzapine for ARFID treatment. In Brewerton and D’Agostino’s study [30], it was demonstrated that for 9 patients, the use of low-dose olanzapine adjunctive to other treatment modalities may have improved patients’ appetite and weight gain, and helped reduce symptoms of anxiety and depression. Regarding cyproheptadine, there is little published support for its use in patients with ARFID, although published reports predating ARFID’s introduction in the DSM-5 have suggested some benefit in younger children with feeding difficulties [31, 32].

This paper describes six cases of children and adolescents with ARFID who were treated by a senior psychiatrist and AM physician at a tertiary care hospital’s specialized ED program, using a combination of medical monitoring, family therapy (using FBT principles but adapted for ARFID), medication (including olanzapine, fluoxetine and in two cases cyproheptadine), and where indicated, cognitive behavioural therapy. Upon reflection, family therapy using ‘Maudsley’ FBT principles, in combination with pharmacotherapy, seems well suited to the treatment of underweight children and adolescents with ARFID, given that FBT for anorexia nervosa focuses on lifting blame, raising anxiety about the dangers of low weight and malnutrition in young people, and empowering parents to take charge of nutrition and to focus on the goal of weight gain. The shared elements in the family therapy sessions for these six ARFID cases include providing psychoeducation to the families about the effects and negative consequences of low weight and insufficient nutrition, raising anxiety about the seriousness of the problem and the need for weight gain, lifting guilt or blame in all family members, empowering parents/caregivers to stand up to the illness and to take charge of nutrition, providing empathy and compassion for the patient, helping the parents to empathize with their child’s pain, fear or discomfort while still being firm about the need to take the nutrition, and focusing the family on issues relating to intake and weight gain, often with detailed intake inventories and the use of weight graphs. Certain other elements of therapy depended on the particular ‘subtype’ of ARFID or the particular details of the case. For example, a child with a severe choking phobia was given cognitive behavioural therapy including psychoeducation about anxiety and phobias, a hierarchy of feared foods, and gradual exposure therapy. Children with severe anxiety at school were taken out of school until weight was at least partially restored, and measures were taken to make the school environment less stressful. Where indicated, individual therapy was sometimes added to the family sessions. In all six of the cases, co-morbid anxiety seemed to affect intake and impair treatment progress. As a means of attenuating this severe distress, augmented treatment for anxiety using psychotherapy (for 5 of the 6 cases) and pharmacotherapy (olanzapine and/or an SSRI, for all 6 cases) was required in conjunction with family therapy to meet the needs of patients.

Further research is required to better understand which treatments or combinations of treatments are best for which illness presentations and subtypes, and how specific presentations might predict response to differing treatment modalities. Moving forward, it will be important for researchers studying treatments for pediatric ARFID to design controlled trials that investigate the effectiveness of ‘Maudsley-type’ family therapy, medication, or cognitive behavioural therapy when used alone and not in combination with other treatments, before studying their combinations. It will also be important for investigators to determine which types of patients (e.g. subtypes of ARFID) respond best to which treatment. Finally, while family therapy adapted from FBT for the treatment of pediatric anorexia nervosa seems suitable for children and adolescents with ARFID, other treatment modalities should be studied for adults diagnosed with ARFID.

Limitations of the present report include the fact that multiple treatment modalities were implemented in an uncontrolled manner, and that our sample size is small without a comparator group. In addition, treatment settings were not standardized (e.g. some patients initiated treatment in hospital, while others began as outpatients). Despite these limitations, the relative absence of specific treatment descriptions in the literature highlight the need for case descriptions to provide preliminary evidence for funding of further controlled research trials.

## Conclusions

This paper describes six adolescents with ARFID who were successfully treated with a combination of medication plus family therapy, and where indicated, targeted cognitive behavioural therapy. These cases illustrate the complex and various ways in which young patients with ARFID can present, the illness’ effect on development and mental health, and the positive outcomes associated with weight gain and concurrent treatment for co-morbid anxiety disorders.

## Abbreviations

%TGW: Percentage of Treatment Goal Weight
ADHD: Attention-Deficit Hyperactivity Disorder
AM: Adolescent Medicine
AN: Anorexia Nervosa
ARFID: Avoidant/Restrictive Food Intake Disorder
ASD: Autism Spectrum Disorder
BMI: Body Mass Index
BN: Bulimia Nervosa
DSM-5: Diagnostic and Statistical Manual of Mental Disorders – 5th Edition
ED: Eating Disorder
FBT: Family-Based Therapy
NG: Nasogastric
OCD: Obsessive Compulsive Disorder
SSRI: Selective Serotonin Reuptake Inhibitor
